# Characterization of the complete mitochondrial genome of the tea slug moth, *Iragoides fasciata* (Lepidoptera: Limacodidae)

**DOI:** 10.1080/23802359.2022.2110008

**Published:** 2022-08-26

**Authors:** Hong-Yan Jiang, Shi-Chun Chen, Xiang Hu, Xiao-Qing Wang

**Affiliations:** Tea Research Institute of Chongqing Academy of Agricultural Science, Chongqing, PR China

**Keywords:** Mitochondrial genome, *Iragoides fasciata*, Limacodidae

## Abstract

Moths of the family Limacodidae are major pests that damage tea trees, fruit trees, and forests. The complete mitochondrial genome of *Iragoides fasciata* (Lepidoptera: Limacodidae) was sequenced. The genome was found to be 15,645 bp in size (GenBank accession no. MK250437), including 13 protein-coding genes (PCGs), two ribosomal RNA genes (rRNAs), 22 transfer RNA genes (tRNAs), and a 431 bp A + T-rich region. Nucleotide composition showed a total A + T content of 82.03% with significant AT-bias. All PCGs were found to start with ATN codons and use the canonical stop codons TAA or incomplete T, except for *cox1*, which was found to utilize CGA as a start codon. Phylogenetic relationships were based on the 13 PCGs with 24 moths, showing that *I. fasciata* is more closely related to other slug moths in the family Limacodidae.

The tea slug moth, *Iragoides fasciata* Moore 1888, belonging to the family Limacodidae in the superfamily Zygaenoidea, occurs in Fujian, Guangdong, Guangxi, Hainan, Yunnan, Guizhou, Sichuan, Hunan, Jiangxi, Zhejiang, Jiangsu, Anhui, Hubei, Shaanxi, and other major tea-producing areas in China. In this study, larvae of *I. fasciata* were collected from a tea plantation in Yongchuan (105°53′ E, 29°23′ N, 690 m), Chongqing Province, China, in May 2018, and were identified by morphology. Each specimen was deposited in the Insect Collection of the Tea Research Institute of Chongqing Academy of Agricultural Science, Chongqing, China (voucher CQNKY-LE-03-02-01, Xiaoqing Wang and wangxiaoqing2891@126.com). We obtained the complete mitochondrial (mt) genome of *I. fasciata* using PCR and performed first-generation sequencing.

The complete mt genome of *I. fasciata*, a typical closed circular molecule, was found to be 15,645 bp in size (GenBank accession MK250437), making it the largest mt genome among the known Limacodidae mt genome sequences (Liu et al. 2016; Liu et al. 2017; Peng et al. 2017; Jiang et al. 2019; Bian et al. 2020). The nucleotide composition of the *I. fasciata* mt genome was found to be as follows: A = 6259 (39.98%), C = 1685 (10.76%), G = 1126 (7.2%), and T = 6575 (42%), with a total A + T content of 82.03% that was significantly AT-biased. AT and GC skew were negative, at −0.025 and −0.199, respectively.

The mt genome was found to encode all 37 genes usually found in animal mt genomes, including 13 protein-coding genes (PCGs), 22 transfer RNA genes (tRNAs), and two ribosomal RNA genes (rRNAs). The gene arrangement in the mt genome of *I. fasciata* is consistent with typical Ditrysia (Lepidoptera). We found 19 bp overlaps and 20 intergenic spacers. The A + T-rich region of the *I. fasciata* mt genome is located between the *rrnS* and *trnM*. It is 431 bp in size and has the highest A + T content (94.2%); the conserved motif ‘ATAGA’ and a 19 bp poly-T stretch. All tRNA genes can fold into the typical cloverleaf secondary structure, except *trnS_1_*, which lacks the dihydrouridine (DHU) arm. We found that 12 of the 13 PCGs started with ATN codons, with only *cox1* utilizing CGA to encode arginine, which has the same synapomorphic characteristic of most Lepidoptera species (Chen et al. 2016; Bian et al. 2020). All PCGs used the canonical stop codon TAA, except *cox1-2* and *nad4*, which were found to have an incomplete stop codon T. Such incomplete stop codons are commonly reported and usually can be established using the tRNA punctuation model, though they can also produce functional stop codons in the polyadenylation process (Ojala et al. 1981; Boore 2001; Stewart and Beckenbach 2009).

We analyzed 13 PCGs sequences of mt genomes with maximum-likelihood (ML) method to explore the phylogenetic relationship of *I. fasciata* with the other 23 moths. The mt genome sequence of *Drosophila melanogaster* served as the outgroup. The monophyly of each superfamily was generally well supported in the ML tree. *T. sinensis*, *I. fasciata*, *C. flavescens*, *M. flavescens*, *P. consocia*, and *N. nigrisigna* belong to the family Limacodidae, and are clustered into a monophyletic branch with a 98% bootstrap value. In Limacodidae, genus-level relationships were determined as follows: *Narosa* + ([*Thosea* + *Iragoides*] + [*Parasa* + {*Monema* +* Cnidocampa*}]) (Figure 1).

**Figure 1.**
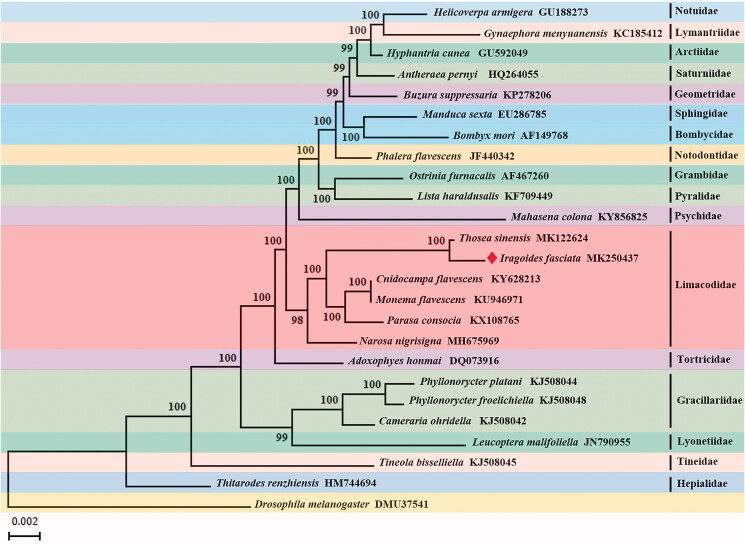
Maximum-likelihood (ML) phylogenetic tree of *Iragoides fasciata* and other moths.

## Ethical approval

This study has been exempted from requiring ethical approval. The Tea Research Institute of the Chongqing Academy of Agricultural Science, Chongqing, China, provided the exemption. Larvae of the slug moth (*Iragoides fasciata*) were collected from a tea plantation in Yongchuan, Chongqing Province, China. It consumes tea leaves, influencing the quality and quantity of tea products. The tea plantation belongs to the Tea Research Institute.

## Author contributions

X.Q. Wang, H.Y. Jiang, and S.C. Chen collected the specimens. H.Y. Jiang, S.C. Chen, and H. Xiang carried out the experiment. H.Y. Jiang wrote the manuscript with support from X.Q. Wang, and S.C. Chen offered data analysis. All authors agree to be accountable for all aspects of the work.

## Data Availability

The data that support the findings of this study are openly available in NCBI’s GenBank at https://www.ncbi.nlm.nih.gov, reference number MK250437.

## References

[CIT0001] Bian DD, Ye WT, Dai ML, Lu ZT, Li MX, Fang YL, Qu JW, Su WJ, Li FC, Sun HN, et al. 2020. Phylogenetic relationships of Limacodidae and insights into the higher phylogeny of Lepidoptera. Int J Biol Macromol. 159:356–363. 32387615 10.1016/j.ijbiomac.2020.05.023

[CIT0002] Boore JL. 2001. Complete mitochondrial genome sequence of the polychaete annelid *Platynereis dumerilii*. Mol Biol Evol. 18(7):1413–1416. 11420379 10.1093/oxfordjournals.molbev.a003925

[CIT0003] Chen SC, Wang XQ, Wang JJ, Hu X, Peng P. 2016. The complete mitochondrial genome of a tea pest looper, *Buzura suppressaria* (Lepidoptera: Geometridae). Mitochondrial DNA A DNA Mapp Seq Anal. 27(5):3153–3154. 25670030 10.3109/19401736.2015.1007310

[CIT0004] Jiang HY, Chen SC, Peng P, Hu X, Lin Q, Yang PX, Wang XQ. 2019. The complete mitochondrial genome of a slug moth, *Narosa nigrisigna* (Lepidoptera: Limacodidae). Mitochondrial DNA Part B. 4(1):320–321.

[CIT0005] Liu QN, Xin ZZ, Bian DD, Chai XY, Zhou CL, Tang BP. 2016. The first complete mitochondrial genome for the subfamily Limacodidae and implications for the higher phylogeny of Lepidoptera. Sci Rep. 6:35878. 27767191 10.1038/srep35878 PMC5073316

[CIT0006] Liu QN, Xin ZZ, Zhu XY, Chai XY, Zhao XM, Zhou CL, Tang BP. 2017. A transfer RNA gene rearrangement in the lepidopteran mitochondrial genome. Biochem Biophys Res Commun. 489(2):149–154. 28546004 10.1016/j.bbrc.2017.05.115

[CIT0007] Ojala D, Montoya J, Attardi G. 1981. tRNA punctuation model of RNA processing in human mitochondria. Nature. 290(5806):470–474. 7219536 10.1038/290470a0

[CIT0008] Peng SY, Zhang Y, Zhang XC, Li Y, Huang ZR, Zhang YF, Zhang X, Ding JH, Geng XX, Li J. 2017. Complete mitochondrial genome of *Cnidocampa flavescens* (Lepidoptera: Limacodidae). Mitochondrial DNA B Resour. 2(2):534–535. 33473889 10.1080/23802359.2017.1365651 PMC7799976

[CIT0009] Stewart JB, Beckenbach AT. 2009. Characterization of mature mitochondrial transcripts in *Drosophila*, and the implications for the tRNA punctuation model in arthropods. Gene. 445(1–2):49–57. 19540318 10.1016/j.gene.2009.06.006

